# A Sodium Leak Current Regulates Pacemaker Activity of Adult Central Pattern Generator Neurons in *Lymnaea Stagnalis*

**DOI:** 10.1371/journal.pone.0018745

**Published:** 2011-04-19

**Authors:** Tom Z. Lu, Zhong-Ping Feng

**Affiliations:** Department of Physiology, Faculty of Medicine, University of Toronto, Toronto, Ontario, Canada; Mount Sinai School of Medicine, United States of America

## Abstract

The resting membrane potential of the pacemaker neurons is one of the essential mechanisms underlying rhythm generation. In this study, we described the biophysical properties of an uncharacterized channel (U-type channel) and investigated the role of the channel in the rhythmic activity of a respiratory pacemaker neuron and the respiratory behaviour in adult freshwater snail *Lymnaea stagnalis*. Our results show that the channel conducts an inward leak current carried by Na^+^ (I_Leak-Na_). The I_Leak-Na_ contributed to the resting membrane potential and was required for maintaining rhythmic action potential bursting activity of the identified pacemaker RPeD1 neurons. Partial knockdown of the U-type channel suppressed the aerial respiratory behaviour of the adult snail *in vivo*. These findings identified the Na^+^ leak conductance via the U-type channel, likely a NALCN-like channel, as one of the fundamental mechanisms regulating rhythm activity of pacemaker neurons and respiratory behaviour in adult animals.

## Introduction

The rhythmic activities of the central pattern generator neurons (CPG) are essential for numerous biological functions, including brain development [1], [2], locomotion [3], energy balance [4], and respiration [5], [6]. The true CPG pacemaker neurons are capable of generating intrinsic bursting rhythms in dependent of synaptic input. One conserved mechanism that is a prerequisite for spontaneous rhythmic activity of pacemaker neurons is regulation of the resting membrane potential (RMP). K^+^ leak has been the classical mechanism to describe regulation of the RMP [7]; however the highly depolarized membrane potential of many pacemaker neurons suggests additional current components [4], [5], [8], [9]. The principles of rhythm generation and its modulation are conserved across species [10], [11].

The great pond snail, *Lymnaea stagnalis* (*L. stagnalis*), is a bimodal breather [12] and its aerial respiratory activity can be easily described by measuring frequency and opening duration of the respiratory gas-exchange orifice (pneumostome). The aerial respiration of *L. stagnalis* is controlled by a simple well-described rCPG network consisting of three large identified neurons [13], including one intrinsic pacemaker neuron, the right pedal dorsal 1 (RPeD1), that initiates rCPG rhythmic activity [13], [14]. The pacemaker neuron RPeD1 exhibits rhythmic activity characterized by intermittent action potential bursts [10], [15]. *L. stagnalis* thus has been used as an animal model to study rCPG properties and regulation [10], [15], [16].

**Figure 1:**
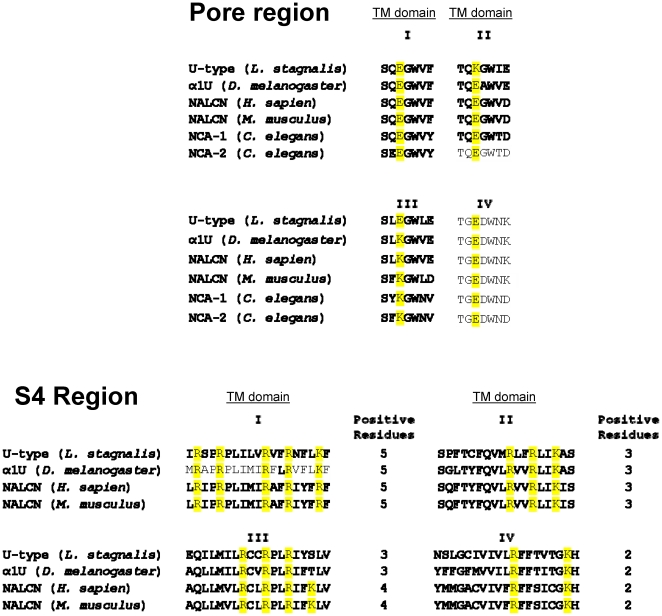
Protein sequence alignments of the U-type pore and S4 regions with the NALCNs. Regional protein sequences of the U-type channel from *Lymnaea stagnalis* (GenBank AAO85435 and AAO84496) were aligned with NALCN channel from *Homo sapien* (GenBank NP_443099) and Mus *musculus* (GenBank NP_796367), NCA-1 (GenBank NP_741413) and NCA-2 (GenBank NP_498054) from *Caenorhabditis elegans*, and α1U from *Drosophila melanogaster* (GenBank AY160083). Positive residues of the S4 region are highly conserved across different species. Pore forming sequence shows high degree of homology with a notable switch between the transmembrane domain II and III.

A putative ion channel (GenBank accession numbers, AF484086 and AF484085) has been partially cloned from *L. stagnalis* and named the U-type channel for an unknown voltage-gated cation channel [17]. Our protein sequence alignment [18] showed that the pore region this uncharacterized putative ion channel has a 55% identity with the non-selective cation channel conducting Na^+^ leak current, NALCN (Sodium Leak Channel Non-selective) [9] of mouse (GenBank: NP_796367) and human (GenBank: NP_443099), and 56% or 45% identity with the NALCN orthologue of *D. melanogaster* (GenBank: AAN77520), and *C. elegans* isoforms NCA-1 (GenBank: NP_741413) and NCA-2 (GenBank: NP_498054), respectively. Specifically, this U-type channel has high homology in the pore and S4 region to its orthologues (**Figure 1**). Therefore, we hypothesize that the U-type channel exhibits similar biophysical properties to its orthologues, NALCN channels, and regulates the RMP [9].

In this study, we investigated the biophysical properties and involvement of the U-type channel in the rhythmic activity of the rCPG pacemaker RPeD1 neuron, and in the aerial respiratory behaviour of the snail, using an RNAi gene silencing approach combined with electrophysiological recordings.

## Results

### The U-type channels regulate the resting membrane potential and are a prerequisite for RPeD1 pacemaker activity

**Figure 2:**
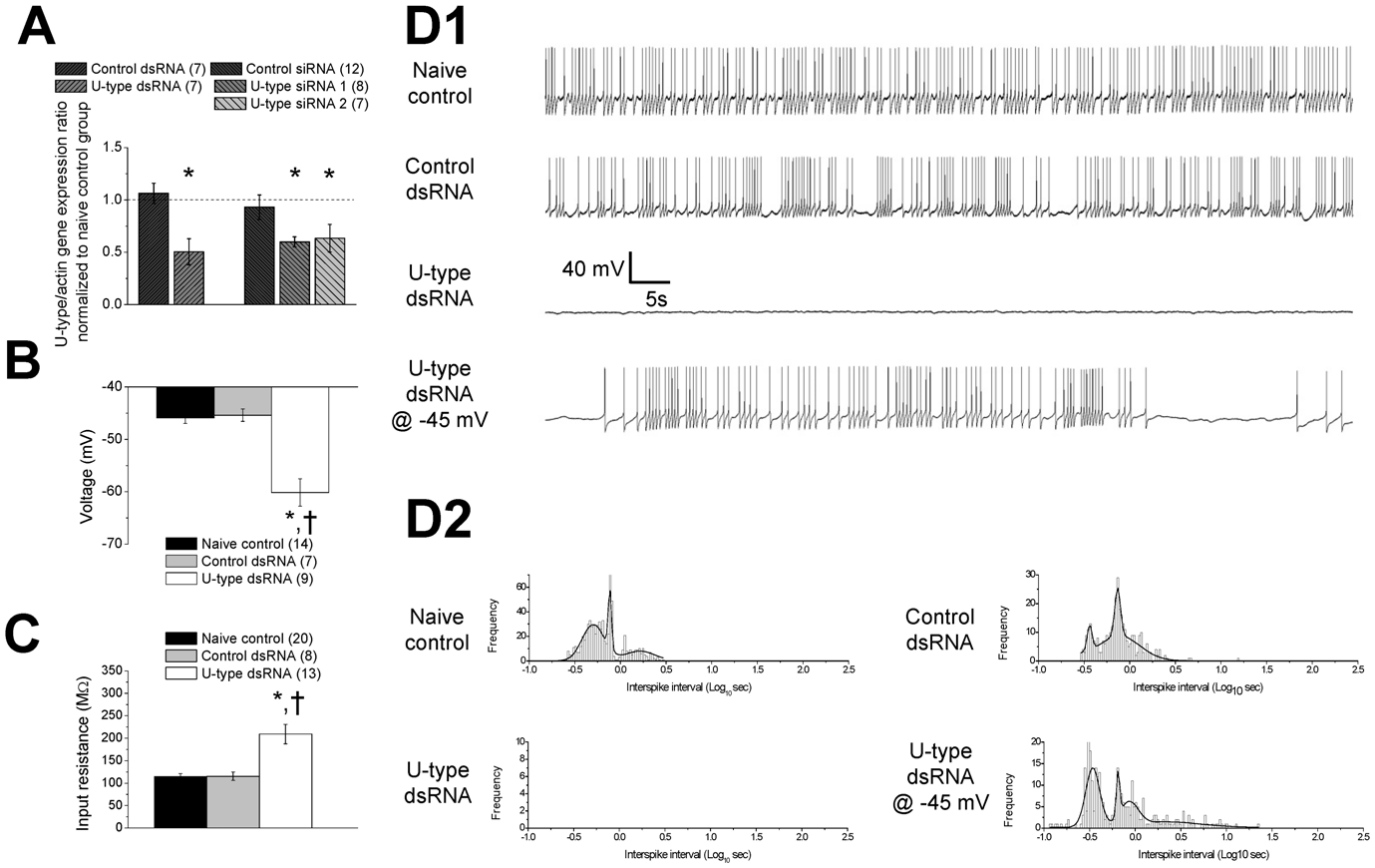
Effects of the U-type dsRNA on rhythmic firing and intrinsic membrane properties in RPeD1 neurons. (A) U-type specific dsRNA/siRNA *in vivo* knockdown was confirmed with real-time qPCR analysis. Expression ratio of the U-type channel to β-actin mRNAs in different experimental groups was normalized to naïve control ratio: control dsRNA (n = 7), U-type dsRNA (n = 7), control siRNA (n = 12), U-type siRNA 1 (n = 8), and U-type siRNA 2 (n = 7). * indicate significant difference (P<0.05) to the corresponding control dsRNA or control siRNA treatment. Isolated individual RPeD1 neurons were maintained in culture in conditioned media (CM), CM + control dsRNA, or CM + U-type dsRNA, and recording was conduced within 24 hours following isolation. (B) Average resting membrane potentials of naïve control (n = 14), control dsRNA (n = 7), and U-type dsRNA (n = 9) treated neurons recorded 2 min after impaling cells. (C) Average input resistance of naïve control (n = 20), control dsRNA (n = 8), and U-type dsRNA (n = 13) treated neurons. (D1) Representative action potential traces of naïve control, control dsRNA, and U-type dsRNA pre-treated neurons recorded at resting membrane potentials, and U-type dsRNA pre-treated neuron depolarized to −45 mV. (D2) Distribution curves of inter spike durations for naïve control neurons (n = 10), control dsRNA neurons (n = 4), U-type dsRNA neurons (n = 5), and U-type dsRNA neurons (n = 5) depolarized to −45 mV. Total inter-spike count is 940 in naïve control, 346 in control dsRNA, 0 in U-type dsRNA, and 304 in U-type dsRNA depolarized to −45 mV. Distributions were best fitted with 4 terms Gaussian curve. All significant difference (P<0.05) between naïve control and U-type dsRNA treatment is denoted by *. All significant different (P<0.05) between control dsRNA and U-type dsRNA treatment is denoted by †.

To determine whether U-type channels are involved in regulating the RMP, we took advantages of the siRNA gene silencing approach to reduce the expression level of U-type channels, as described previously [19]–[21]. We first determined the efficiency of the acute knockdown of U-type channel expression. Real-time PCR analysis (**Figure 2A**) showed that ganglionic expression level of U-type mRNA transcripts was reduced by ∼50% when either dsRNA or either siRNA specific to the U-type gene was applied *in vivo* for 3–4 days. We then isolated the respiratory pacemaker neuron, RPeD1, from whole animals that were injected with control dsRNA/siRNA or U-type dsRNA/siRNA for 3 days, and cultured these neurons in respective RNAi treatments overnight. Intracellular sharp electrode recordings from these treated cells show that the RMP, recorded with zero current injection (**Figure 2B**) was more hyperpolarized and input resistance (**Figure 2C**) increased in the U-type dsRNA group relative to those of the controls. These data suggest that the U-type channel may conduct current that is required to maintain the RMP at the more depolarized potential.

To test indeed that the U-type channel regulates the pacemaker activities, we compared firing patterns of the spontaneous action potentials in control and U-type knockdown preparations. The distribution of the durations between action potentials (intra-spike interval) over 20 minutes of recordings were analyzed to characterize the burst firing pattern. Naïve control and control RNA groups (**Figure 2D2**) have similar spike patterns and the inter-spike intervals in the isolated RPeD1 cells showed two populations of shorter intervals (0.40 s, and 0.79 s) in the control groups. Surprisingly, no spontaneous activity was observed at resting membrane potential in all the U-type dsRNA treated cells (**Figure 2D1**). To test whether these activity patterns differed between groups because of the more hyperpolarized membrane potential of RPeD1 neurons in the U-type dsRNA group, we injected a compensatory current to depolarize the membrane potential to −45 mV, thereby approximating the RMP of the control cells (**Figure 2B**). Interesting, the U-type pre-treated neurons resumed rhythmic firing (**Figure 2D1**), albeit with a different rhythmic distribution (**Figure 2D2**). In contrast, the AP properties, including the AP amplitude, rise constant, decay constant, and half-width constant, were similar between the U-type dsRNA and the control groups (data no shown). The results demonstrated that U-type expression within the pacemaker neuron, RPeD1, affected spontaneous rhythmic activity.

### U-type channels conducted an inward Na^+^ leak current at hyperpolarizing voltages

**Figure 3:**
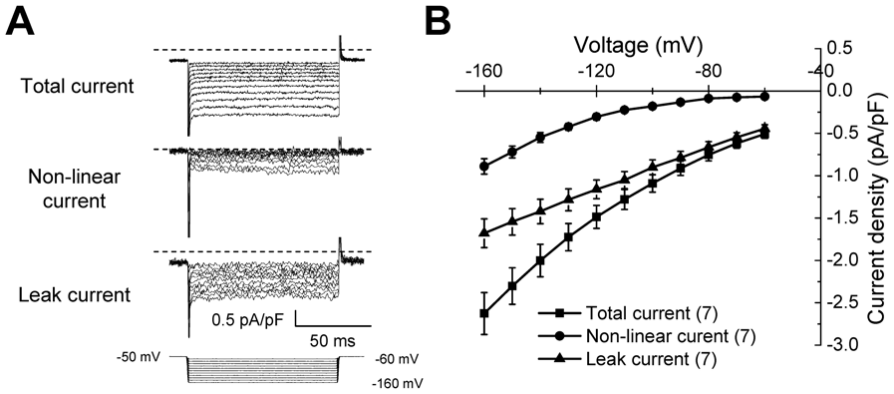
Whole-cell current in individual RPeD1 neurons isolated from naïve control animals. (A) Representative hyperpolarizing inward current density traces of the total current, non-linear voltage-dependent current and inward leak currents from one individual RPeD1 neuron. (B) Average current density-voltage (I–V) relation of the total current (n = 7), the non-linear current (n = 7) and the leak current (n = 7) recorded from seven RPeD1 neurons. All data were represented as mean ± S.E.M.

**Figure 4:**
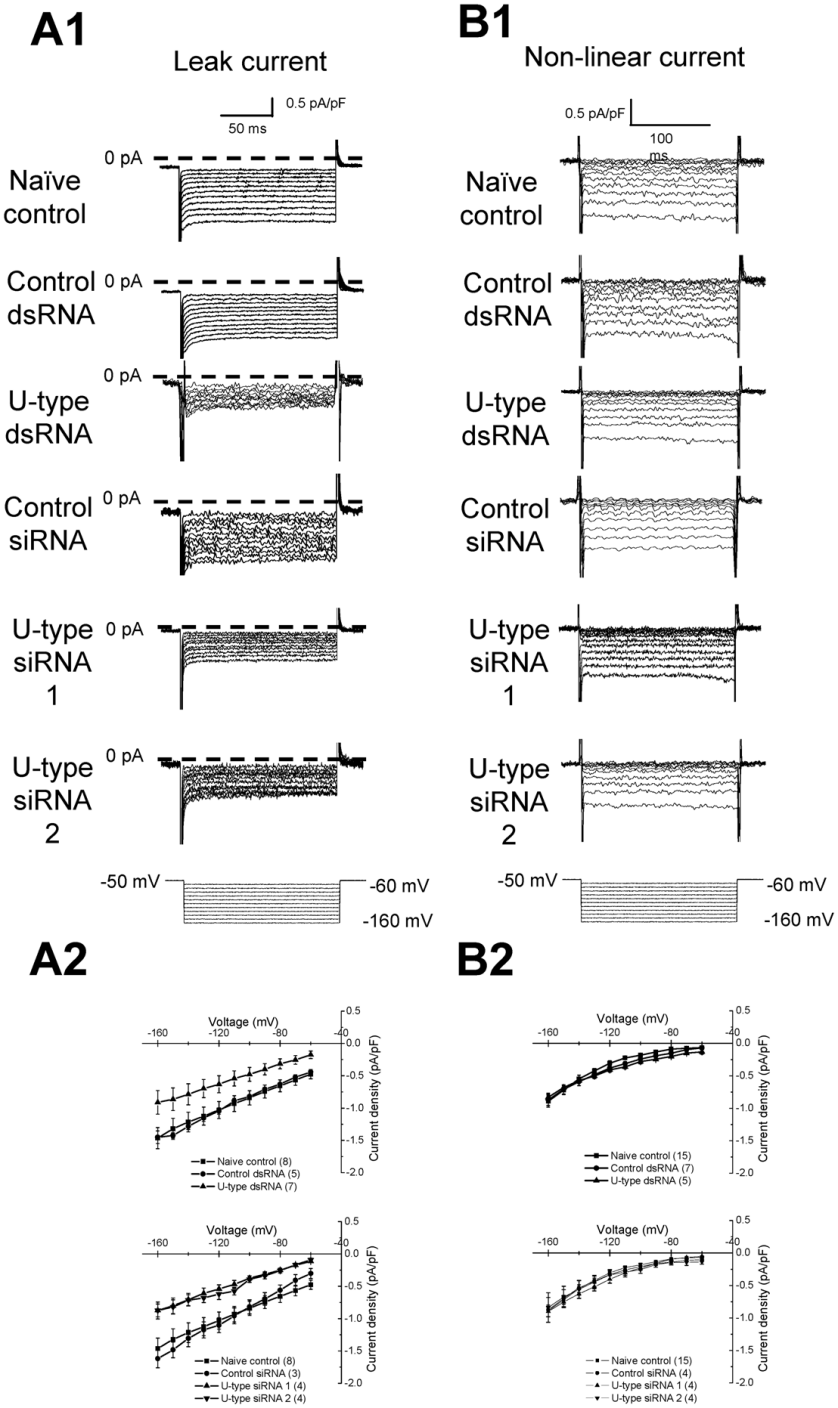
U-type RNAi knockdown reduces inward hyperpolarizing leak currents in RPeD1 neurons. U-type RNAi treatment reduces inward hyperpolarizing I_leak_. (A1) Representative I_Leak_, and (A2) average current density-voltage (I–V) relation of the hyperpolarizing inward I_Leak_ in RPeD1 cells from naïve control (n = 8), control dsRNA (n = 5), U-type dsRNA (n = 7), control siRNA (n = 3), U-type siRNA 1 (n = 4), and U-type siRNA 2 (n = 4) treated RPeD1 neuron. I_Leak_ at all hyperpolarizing voltages reduced significantly in the U-type dsRNA/siRNAs groups as compared to the control groups (P<0.05). (C) Non-linear leak component was not significantly affected by the U-type RNAi treatment. (B1) Representative non-linear component, and (B2) average current density-voltage relations of the non-linear component for naïve control (n = 15), control dsRNA (n = 7), U-type dsRNA (n = 5), control siRNA (n = 4), U-type siRNA 1 (n = 4), and U-type siRNA 2 (n = 4).

Major determinant of the RMP is the leak conductance. To investigate whether U-type channels conduct a leak current, we first established a two-step recording protocol in a whole-cell configuration to segregate a linear leak current from the voltage-dependent hyperpolarizing current. The representative recordings in **Figure 3** show that the inward hyperpolarizing current (total current) recorded from RPeD1 contained two components; a non-linear and a linear leak current. The total hyperpolarizing current was initially recorded at hyperpolarizing step voltages in order to limit activation of major voltage-gated channels (**Figure 3A**). The non-linear hyperpolarizing current was recorded at the identical voltage steps with an online leak-subtraction protocol. The linear leak current (I_Leak_) component was obtained by subtracting the non-linear current from the total current. The current density-voltage relations for the total, non-linear and linear currents are shown in **Figure 3B**. The two components of the total hyperpolarizing current are clearly separated by the recording protocols (**Figure 3**). We then used the established protocol to determine whether I_Leak_ is conducted by U-type channels in RPeD1 neurons. As shown in the representative recordings of **Figure 4A1**, the leak current was reduced in the RPeD1 cells pre-treated with U-type channel dsRNA/siRNAs. The current - voltage (I–V) relation of the I_Leak_ conductance of U-type dsRNA treated RPeD1 neurons was significantly reduced at all the tested voltage-steps, relative to both the untreated naïve controls and the control dsRNA treated neurons (**Figure 4A2**). Similar results were observed in U-type siRNA treated groups. In contrast, the non-linear hyperpolarizing current in RPeD1 neurons was not affected by RNAi treatment (**Figure 4B1** and **B2**). These findings indicate that the U-type channel contributes in part to the I_Leak_ component of the RPeD1 cells at hyperpolarizing voltages.

**Figure 5:**
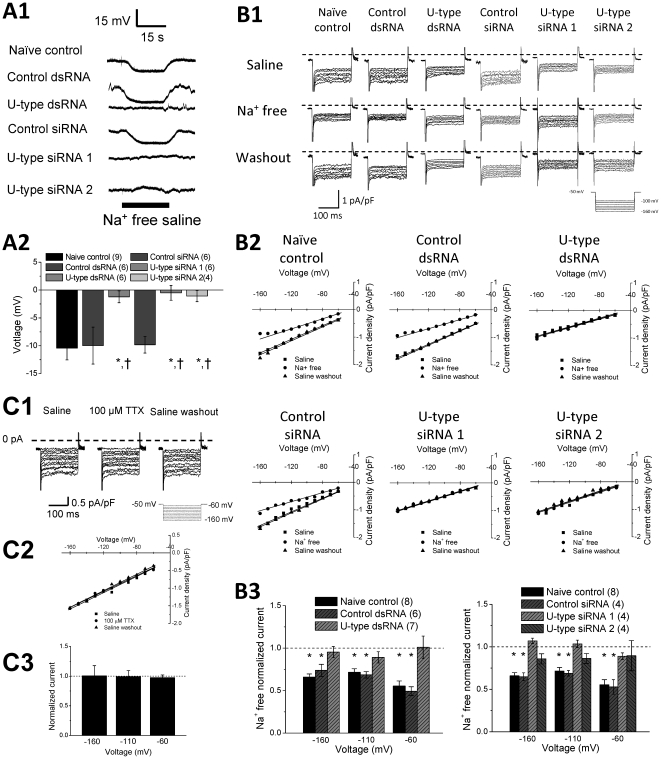
I_Leak_ conducted by the U-type channel in RPeD1 cells is carried by Na^+^ ions. (A1) Representative voltage traces of naïve control, control dsRNA/siRNA, and U-type dsRNA/siRNA 1 & 2 with and without Na^+^ free saline perfusion. (A2) Average voltage difference between Na^+^ free perfusion and saline perfusion for naïve control (n = 9), control dsRNA (n = 6), U-type dsRNA (n = 6), control siRNA (n = 6), U-type siRNA 1 (n = 6), U-type siRNA 2 (n = 4). (B1) Representative I_Leak_ in RPeD1 cells from naïve control, control dsRNA/siRNA, and U-type dsRNA/siRNA 1&2, at hyperpolarizing voltages in saline or Na^+^ free solution (B2) Representative I_Leak_ density-voltage (I–V) relations of naïve control, control dsRNA/siRNA, and U-type dsRNA/siRNA 1&2 in saline or Na^+^ free solution. (B3) comparison of the average normalized I_Leak_ in Na^+^ free solution over saline at −160 mV, −110 mV, and −60 mV for naïve control (n = 8), control dsRNA (n = 6), U-type dsRNA (n = 7), control siRNA (n = 4), U-type siRNA 1 (n = 4), and U-type siRNA 2 (n = 4). A significant reduction in normalized I_Leak_ under Na^+^ free condition was observed in RPeD1 cells from the naïve control and control dsRNA/siRNA groups, but not from U-type dsRNA/siRNA groups. All significant difference (P<0.05) between Na^+^ free saline current normalized to saline is denoted by *. The dashed line represents the current activity in saline. (C) Application of 100 µM TTX did not significantly affect I_leak_. (C1) Representative leak current traces of naive control neurons in saline, 100 µM TTX, and saline wash under a hyperpolarizing step voltage protocol. (C2) Representative current-voltage relation for the leak current observed in saline, 100 µM TTX, and saline wash. Line represents a linear fit. (C3) Summary of relative current density of 100 µM TTX normalized to that in saline at various hyperpolarizing voltages: −160 mV (n = 5), −110 mV (n = 5) and −60 mV (n = 5). No significant difference was observed in the normalized currents among all the groups.

The reversal potential of our observed leak current was not at −70 mV (approximate reversal potential of K^+^), suggesting additional contributing components. Therefore, we next tested whether Na^+^ is conducted through U-type channel using an ion substitution approach. We first determine whether U-type channels conduct a Na^+^ current that contribute to membrane potential regulation, by measuring changes in the membrane potential in a Na^+^-free bath solution. As shown in **Figure 5A1**, the membrane potential became more hyperpolarized when Na^+^ was substituted with equimolar NMDG^+^ in all the control groups, but no significant change was observed in the U-type dsRNA or siRNA groups. The data are summarized in **Figure 5A2**. A ∼10 mV difference in the membrane potential was observed from the control cells in Na^+^-free condition which was in agreement with the change in the resting membrane potential after dsRNA or siRNA treatment (**Figure 2B**). We then used whole-cell (ruptured) recording to identify whether Na^+^ current through U-type channel contributes to the ILeak. As shown in representative current recordings of **Figure 5B1** and I–V relation curves of **Figure 5B2**, the I_Leak_ recorded at the hyperpolarizing voltages decreased in the naïve control, control dsRNA, and control siRNA treated RPeD1 neurons, when Na^+^ in the bath solution was substituted with equimolar NMDG^+^. In contrast, the current recorded with the same hyperpolarizing protocol in the U-type channel dsRNA/siRNA groups was not affected by the Na^+^-free solution. The data are summarized in **Figure 5B3** that the leak conductance was significantly reduced in Na^+^-free solution (P<0.05) only in the control groups, but not in the U-type channel knockdown groups. We compared the Na^+^ current components by subtracting the current recording in Na^+^-free condition from the total Na^+^ current in saline between the controls and the U-type knockdown groups. We found that U-type knockdown significantly reduced the inward hyperpolarizing Na^+^ currents, further confirming that a large component of the Na^+^ current was conducted by the U-type channels (**Figure S1**). To test whether the Na^+^ leak current is sensitive to tetrodotoxin (TTX), a selective blocker of voltage-gated sodium channel, we first studied the IC_50_ value of the voltage-gated sodium channels in RPeD1 neurons to TTX. An IC_50_ value of ∼25 mM TTX was observed (**Figure S2**), which was in a good agreement with that observed in the cerebral giant cells of *L. stagnalis* [22], indicating the snail channels are intrinsically less sensitive to TTX block as compared with the mammalian channels. To ensure sufficient TTX concentration, 100 µM TTX (∼4 fold of the IC_50_ value for the snail voltage-gated Na channels) was perfused, however no change was observed in the I_Leak_ (**Figure 5C**). These findings indicate that the U-type channel carries a TTX-insensitive leak Na^+^- current (I_Leak-Na_) in RPeD1 cells, which directly regulates the RMP.

### U-type channel conductance is pharmacologically similar to reported NALCN channel conductance

**Figure 6:**
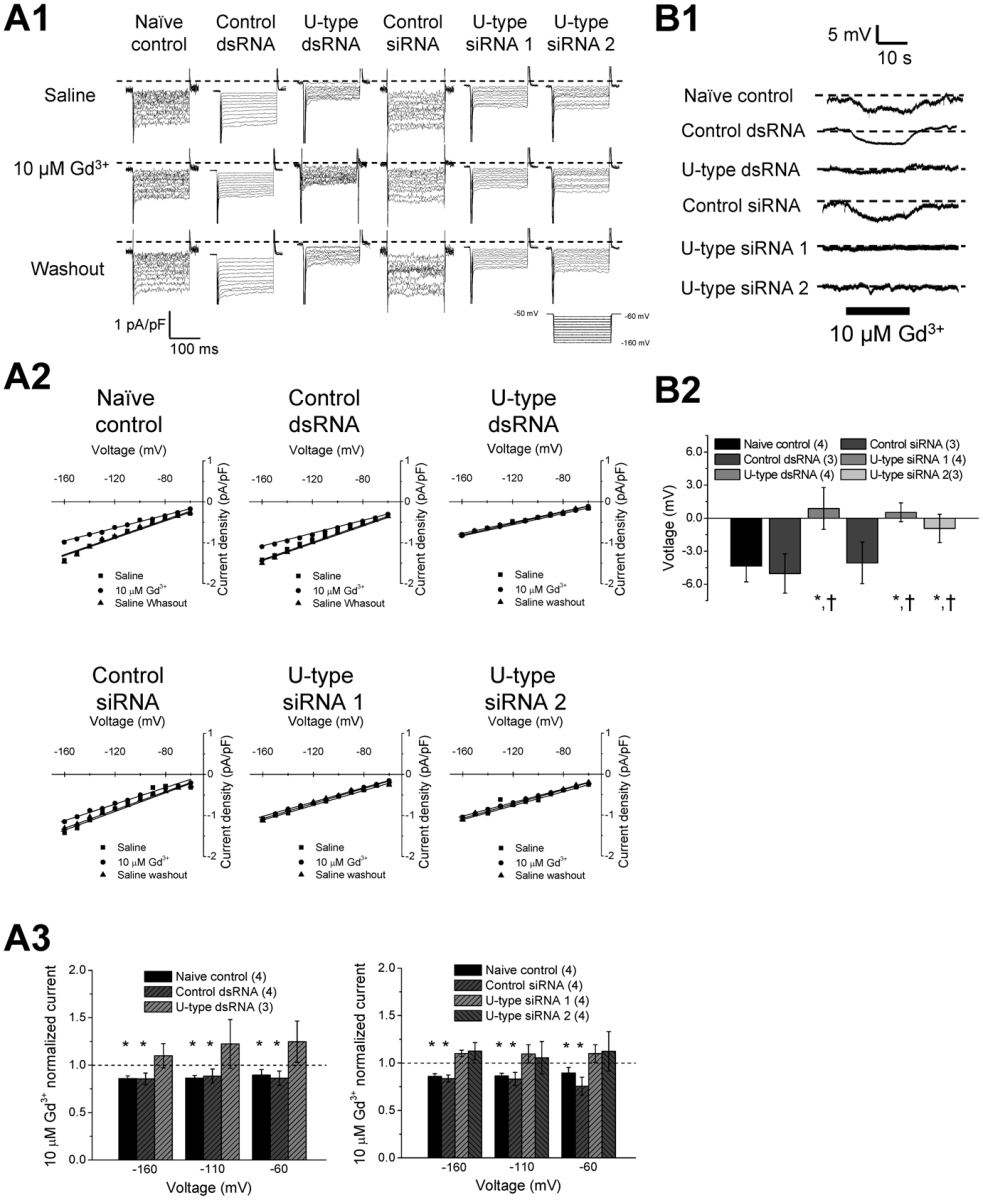
Gd^3+^ partially blocked I_Leak_ via the U-type channels in RPeD1 neurons. (A) I_Leak_ conducted by the U-type channel is partially blocked by Gd^3+^. Representative I_Leak_ (A1) and I_Leak_ - voltage (I–V) relations (A2) of naïve control, control dsRNA/siRNA, and U-type dsRNA/siRNA 1&2 in saline with or without 10 µM Gd^3+^. (B3) Average normalized I_Leak_ in 10 µM Gd^3+^ over saline at -160 mV, −110 mV, and −60 mV for naïve control (n = 4), control dsRNA (n = 4), U-type dsRNA (n = 3), control siRNA (n = 4), U-type siRNA 1 (n = 4), and U-type siRNA 2 (n = 4). The dashed line represents the current activity in saline. 10 µM Gd^3+^ reduced significantly the I_Leak_ in the naïve control and control dsRNA/siRNA groups, but not in U-type dsRNA/siRNA groups. Data are presented as mean ± SEM. Significant difference (P<0.05) between Na^+^ free saline current normalized to saline is denoted by *. (B) 10 µM Gd^3+^ hyperpolarized the membrane potential. (B1) Representative records of the membrane potentials of RPeD1 from naïve control, control dsRNA/siRNA, and U-type dsRNA/siRNA group. 1 & 2 indicate perfusion with and without 10 µM Gd^3+^, respectively. (B2) Average difference of the membrane potential by 10 µM Gd^3+^ in naïve control (n = 4), control dsRNA (n = 3), U-type dsRNA (n = 4), control siRNA (n = 3), U-type siRNA 1 (n = 4), U-type siRNA 2 (n = 3) groups. * and † indicate the significant difference (P<0.05) between naïve control and U-type RNAi treatment and between control RNAi and U-type RNAi treatment, respectively.

An I_Leak-Na_ is conducted by NALCN channels at rest [9]. These channels are non-sensitive to TTX [9], and can be partially blocked with Gd^3+^ [9] and activated by low [Ca]_o_ [23]. We thus asked whether our observed TTX-insensitive I_Leak_ has similar pharmacological properties to NALCN channels. We first tested whether Gd^3+^ applications would affect I_Leak_. As shown the representative recordings in **Figure 6A1** and I–V curves in **Figure 6A2**, 10 µM Gd^3+^ suppressed the I_Leak_ in the control groups, but with minimal effect in U-type dsRNA/siRNA treated groups. Gd^3+^ significantly reduced the normalized leak current in the groups (P<0.05), but slightly enhanced the current in the U-type RNAi groups (summarised in **Figure 6A3**). The Gd^3+^-blocked current component in the control groups was smaller (**Figure 6A3**) than the Na-dependent leak current component (**Figure 5B3**); therefore, our findings indicate that Gd^3+^ only partially blocks the I_Leak-Na_ conducted by U-type channel. To further confirm that a partial blockade by Gd^3+^ affects the functional properties of U-type channel, we measured the membrane potential of RPeD1 in saline containing 10 µM Gd^3+^. The membrane potential in the control cells was reduced by ∼5 mV in Gd^3+^ condition (**Figure 5A**) but was not affected by Gd^3+^ in U-type knockdown group, consistent with our voltage-clamp data showing that Gd^3+^ partially blocked leak current of RPeD1.

**Figure 7:**
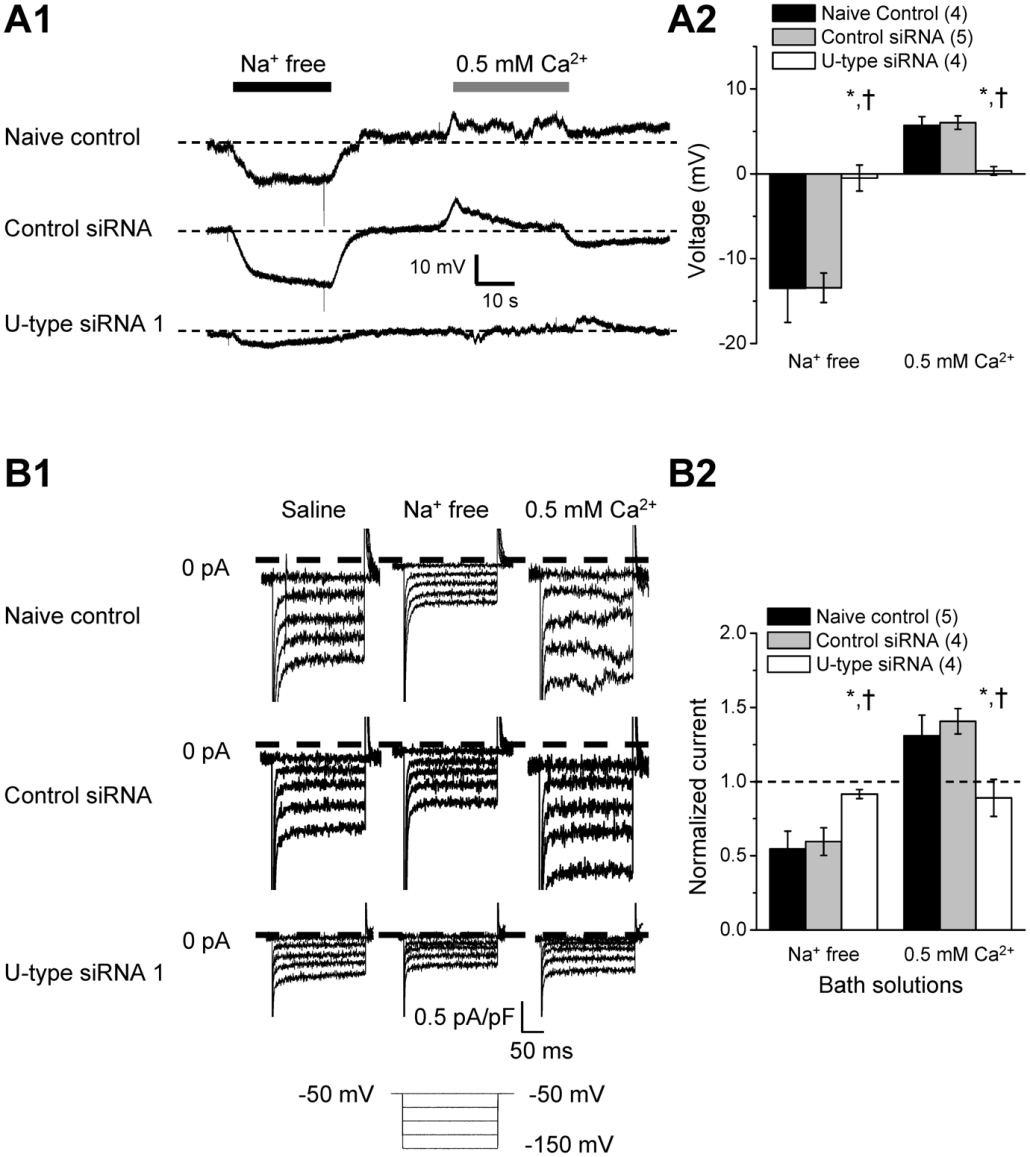
Low extracellular Ca^2+^ depolarizes the membrane potential by enhancing U-type channel activity in RPeD1 neurons. (A) U-type channel knockdown prevents low extracellular Ca^2+^-induced membrane depolarization. (A1) Representative voltage traces of naive control, control siRNA, and U-type siRNA treated RPeD1 neurons perfused with saline, Na^+^ free or low Ca^2+^ saline. Partial U-type knockdown reduces membrane hyperpolarization by Na^+^ free solution or depolarization by 0.5 mM Ca^2+^. (A2) Average voltage differences of naive control (n = 4), control siRNA (n = 5), and U-type siRNA (n = 4) treated RPeD1 under Na^+^ free or 0.5 mM Ca^2+^ perfusion. (B) Low extracellular Ca^2+^ increased I_leak_ is reduced in U-type knockdown. (B1) Representative I_leak_ traces of naive control, control siRNA, and U-type siRNA treated RPeD1 in saline, Na^+^ free, and 0.5 mM Ca^2+^ solution. (B2) Average I_Leak_ normalized to saline of naive control (n = 5), control siRNA (n = 4), and U-type siRNA (n = 4) treated RPeD1 in Na^+^ free, and 0.5 mM Ca^2+^ at −100 mV. The dashed line represents the current activity in saline. Data are presented as mean ± SEM. * and † indicate significant difference (P<0.05) between naïve control and U-type RNAi treatment and control RNAi and U-type RNAi treatment, respectively.

We then test whether the U-type channel properties are affected by low [Ca]_o_. As shown the representative recordings in **Figure 7A1** and I–V curves in **Figure 7A2**, 0.5 mM [Ca]_o_ depolarized the membrane potential in the control groups, but with a minimal effect in U-type dsRNA/siRNA treated groups. The low [Ca]_o_ also significantly enhanced the normalized leak current in the control groups (P<0.05), but with no significant effect on the current in the U-type RNAi groups (**Figure 7B**). Taken together, our data suggest similar pharmacological properties between U-type channels and NALCN channels.

### Partial U-type channel knockdown reduced the aerial respiratory behaviour in adult animal in vivo

**Figure 8:**
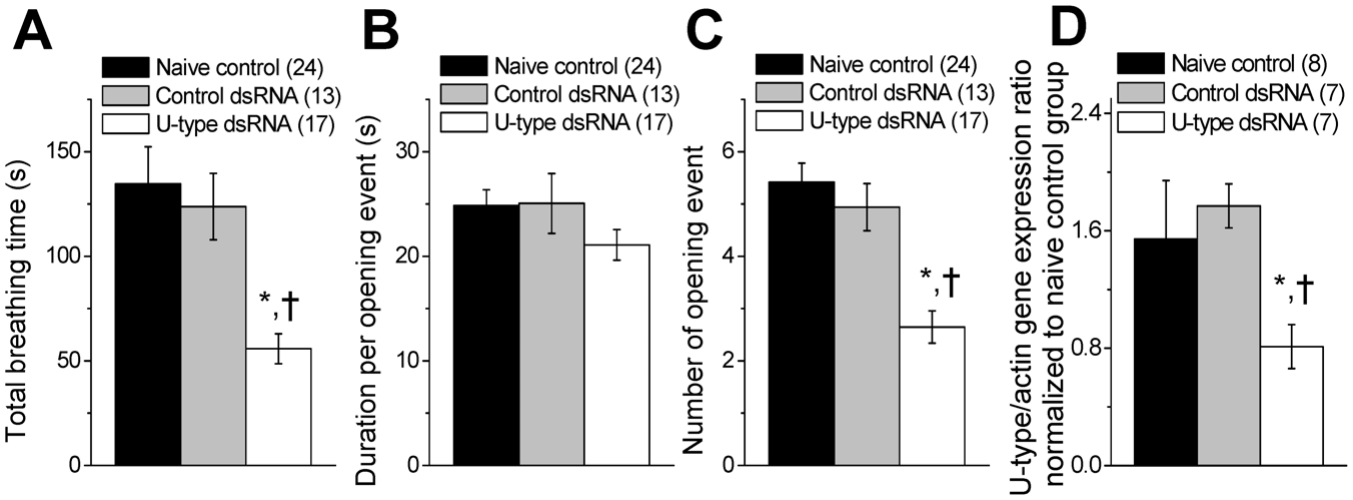
Acute U-type dsRNA knockdown suppresses aerial respiratory behaviour in adult *L. stagnalis in vivo*. Summary of the (A) total breathing time, (B) duration per opening event, and (C) number of opening events for naïve control (n = 24), control dsRNA (n = 13), and U-type dsRNA injected snails (n = 17). U-type dsRNA injected snails only showed significant reduction in total breathing time (55.82±7.13 s) and number of opening events (2.65±0.31) when compared to naive control (total breathing time of 134.60±17.76 s and 5.42±0.36 opening events) and control dsRNA (total breathing time of 123.77±15.82 s and 4.94±0.45 opening events). Average opening duration for U-type dsRNA (21.09±1.46 s) injected group is not significantly different from the naive control (24.85±1.51 s) and control dsRNA (25.05±2.86 s) injected snails. (D) Summary of real-time PCR performed following behavior experiments. Ganglia from U-type dsRNA injected snails that showed decrease in aerial respiratory behavior were used to confirm U-type channel knockdown with real-time qPCR. The result was then compared with that of naive control and control dsRNA groups. Beta-actin control gene was used for ratiometric analysis of U-type mRNA expression level. * and † indicate significant difference (P<0.05) from the naïve control and control dsRNA treatments, respectively.

Having established that U-type channel knockdown slows firing rhythmic properties of isolated RPeD1 neurons (**Figure 2**), we next investigated whether the U-type channel plays a role in regulation of respiratory behaviour the adult animal *in vivo*. We measured aerial respiratory activity of the snails from the naïve control, control dsRNA or U-type dsRNA treated groups. **Figure 8A** shows that the total duration of pneumostome opening of the U-type dsRNA group (55.8±7.1 s; n = 17; p<0.05) during the 1 hour observation period was significantly reduced relative to the naïve control (134.60±17.76 s; n = 24) and control dsRNA (123.8±15.9 s; n = 13) groups. Interestingly, the averaged duration of opening event (**Figure 8B**) only slightly decreased in U-type dsRNA group (21.09±1.46 s; P>0.05), whereas the number of opening events (**Figure 8C**) was significantly reduced (2.68±0.31; P<0.05). Acute U-type channel gene knockdown was confirmed using real-time qPCR (**Figure 8D**); U-type specific dsRNA treated animals exhibiting a reduced total breathing time showed significantly low U-type mRNA expression level ratio (0.81±0.15; P<0.05) as compared to the naive control (1.545±0.40) and control dsRNA group (1.77±0.15). These observations support the hypothesis that U-type channel plays an important role in maintaining the respiratory activity in adult animal via regulation of rCPG rhythmic activity by regulating the resting membrane potential of the pacemaker neurons.

## Discussion

In this study, we have demonstrated that an inward Na^+^-conductance leak current component at rest regulates the rhythmic activity of a respiratory pacemaker neuron, RPeD1. Acute knockdown of the channel gene decreased the functional channel expression and suppressed the rhythmic firing of the pacemaker neuron, and inhibited breathing activity. The U-type channel protein sequence shared ∼50% homology with the pore region of the NALCN channel and exhibited similar biophysical and pharmacological properties, thus the U-type channel is likely a *Lymnaea* orthologue of NALCN-like channel. The I_Leak-Na_ conducted though the U-type channels is critical in modulating the rCPG rhythmic activity. This is the first study providing direct evidence of a NALCN-like channel regulating activities of pacemaker neurons, and its functional significance in breathing activity determined by the pacemaker neurons at the whole animal level.

Many ion channels have been identified as contributors to pacemaker activities in vertebrates. These include: persistent Na^+^ currents (I_NaP_) [24]–[28], leak K^+^ current (I_K-LEAK_) [26], Ca^2+^-activated voltage-insensitive non-specific cation current (I_CaN_) [28], [29], hyperpolarizing activated current (I_h_) [30]–[33], small conductance Ca^2+^-activated K^+^ current (I_SK_) [32], [34], [35], and subthreshold Ca^2+^ currents (I_Ca-T_) [27], [36]. In invertebrates, similar currents have also been identified to modulate various CPG network models [11], [22], [37]. In our study, Na^+^ leak through U-type channels (I_U-type_) represents a major component in subthreshold current densities. Background Na^+^ current has been previously identified in respiratory pacemaker neurons in rodent [5], by stabilizing bursting activities. Given the relative depolarized nature of the RMP in rodent rCPG pacemaker neurons [5] and other pacemaker neurons [4], [8], it is likely that the I_leak-Na_ current via NALCN channel regulates the resting membrane potential in the similar manner of U-type channel in the snail. The voltage-dependent component of inward hyperpolarizing current (**Figure 2** and **Figure 3**) is likely an I_h_ conductance. Although previous studies have not identified I_h_ component from other *L. stagnalis* neurons [22], the presence of the I_h_ in RPeD1 cells has not been excluded. Pacemaker bursting activity in pacemaker neurons is also mediated by I_NaP_ and I_K-LEAK_ in rodents [26], [38] and *L. stagnalis* [22]. I_Leak-Na_ via the NALCN or NALCN-like channels could be another conserved component that is essential for bursting activity across species.

The RMP is one of the essential prerequisite that determines pacemaker activity [39], and is determined largely by the ionic permeability of the cell membrane [7], [40] at rest. Membrane permeability to K^+^ through K leak channels has been considered as the key conductance to determine the RMP [7]. However, the RMP of most pacemaker neurons are more depolarized than the equilibrium potential of K^+^, suggesting additional outward current component [4], [8]. Resting Na^+^ conductance may be derived from numerous sources, including window current from voltage-gated Na^+^ channels, hyperpolarization-activated channels, persistent Na^+^ channels and Na^+^-coupled transporters. NALCN channels have been considered to be the major contributor to background Na^+^ leak current in hippocampal neurons [9], [23]. The NALCN channel was first cloned from rat brain preparation [41]. The leak current conductance of the channel, however, remains inconclusive because of a lack of the background Na^+^ leak conductance following overexpression of the NALCN channel on HEK293 cells [42]. Our results in the pacemaker RPeD1 neurons support the notion that NALCN-like channels conducts leak Na^+^ current at rest.

The *in-vivo* knockdown through RNAi gene silencing has been widely used to study protein function in developed organisms [43], including *L. stagnalis* [20], [21], [44]. Both long dsRNA and short siRNA have been used to induce gene silencing in *L. stagnalis* [20], [21], [44]. One of the shortcomings of RNAi approach is the possibility of its off-targeting effect on non-specific genes. We attempted to remedy this possibility by employing three different dsRNA sequences consisting of both long dsRNA and short siRNAs. The two siRNA were synthesized from two different fragments of U-type mRNA sequence (AF484085 and AF484086). The selection criteria consist of selecting for moderate to low G/C content, biasing toward the 3′-terminus and purposely avoiding sequences encoding the transmembrane domain [45]. The resulting siRNA sequence was BLAST searched against known genes sequences in all available databases to avoid complementary paring with any known ion channel sequences. All three RNAi sequences resulted in a >40% knockdown in U-type mRNA level, a reduction of the I_Leak-Na_, hyperpolarized resting membrane potential, decrease in AP firing activity of the pacemaker RPeD1 neuron, and suppressed respiratory behaviour. Our findings are consistent and thus suggest that the U-type channel RNAi in *L. stagnalis* is likely specific.

NALCN channel may affect synaptic transmission. *C. elegans* orthologues of the NALCN channels are critical in the conduction of depolarizing signal from the soma to the axon [46] and mutation of this channel impairs presynaptic release [47]. NALCN orthologue is highly expressed at synapses in *D. melanogaster* [48], but perisynaptically in *C. elegans* [46]. In rodents, the NALCN is modulated by substance P and neurotensin through G-protein independent and Src family of tyrosine kinases-dependent pathway [49]. The NALCN channel conductance in the pancreatic β-cells is activated by M3 muscarinic receptors [42]. The rCPG network in adult *L. stagnalis* consists of three neurons [15]. RPeD1 forms a mutual inhibitory synapse with VD4 neuron which innervates to motor neurons closing pneumostome. RPeD1 forms excitatory synapse with IP3I which leads to pneumostome opening. Reduction of U-type channel expression decreases the number of openings, but has less effect on closing of the pneumostome (**Figure 8**), indicating the rCPG network is differentially regulated by I_Leak-Na_ conductance. Shifting the RMP to the hyperpolarizing direction may reduce the excitatory synaptic input of RPeD1 to IP3I and relieve the inhibitory input on VD4; thus, the rCPG output favours the decrease in pneumostone openings rather than duration of the opening. This study leads to new avenues to further our understanding of rhythm regulation of CPG network.

## Materials and Methods

### Animals and aerial respiratory behavioural observation

Freshwater pond snails, *L. stagnalis*, were obtained from an inbred culture at the Free University in Amsterdam, and raised and maintained in 18–20°C aquaria on a 12 hr light/12 hr dark cycle aquaria at the University of Toronto [20], [50]. Six-week old snails were used in all experiments. To study the aerial breathing behaviour of the snails, individually labelled snails were placed in a 1000 mL beaker filled with 500 mL of water. Snails were allowed 10 minutes acclimatizing to the new environment. Aerial respiratory behaviour of the snails was monitored by observing the physical opening and closing of the gas exchange orifice, pneumostome, at the water-air interphase. The duration and number of each pneumostome opening and closing event were recorded for 1 hour.

### Ganglionic RNA preparation and cDNA synthesis

The central ring ganglia were excised from anaesthetized snails (in 10% v/v Listerine for 5 min). Two excised ganglionic rings were used for each total RNA extraction following a modified Trizol method (Invitrogen) as described preciously [50]. First strand synthesis of cDNA was conducted using SuperScript III reverse transcriptase (Invitrogen) with random hexamer primer (Fermentas) in total volume of 20 µl for 1 µg of total RNA.

### RNAi synthesis and delivery

The double-strained RNA and siRNA were synthesized as described previously [20], [21], [50]. Specifically, the primers for the U-type channel (GenBank#, AF484085) dsRNA were designed with T7 phage polymerase promoters at the 5′ end of the primers, and the primer sequences were shown in Table 1. The U-type channel gene was amplified from a standard DNA library of the snail ganglia, using the 2x PCR master mix (Fermentas, USA) using conventional PCR (PTC-100TM Programmable Thermal Controllor). The samples were amplified with the temperature profile of 94°C/2 min, 94°C/30″, (T_m_ - 5°C)/30″, 72°C/30″ and a final elongation of 72°C for 10 minutes. The amplified products were then purified using the PureLink™ PCR Purification Kit (Invitrogen, USA) according to the manufacturer's instructions. The control dsRNA was synthesized from a linearlized pcDNA3 vector with a T7 and SP6 promoter sequence. The RNA was transcribed using MEGAscript High Yield Transcription Kit following instructions provided by the manufacturer (Ambion). The synthesized RNAs were then denatured in 85°C water bath for 10 minutes and allowed to gradually anneal as bath cooled to room temperature.

**Table 1:** Primer sequences of U-type channel and β-actin for real-time PCR analysis.

Name	Type	Sequence	Expected bp
*U-type T7 (AF484086.1)	Sense	5′-TAATACGACTCACTATAGGGAGAATTGGTGTTGGTCATTGGTACG-3′	319
	Anti-sense	5′-TAATACGACTCACTATAGGGAGACATAACTTCAATCCACCCTTTCTG-3′	
U-type (AF484086.1)	Sense	5′-CCGCAAATGGTTCGACTCTA-3′	129
	Anti-sense	5′-TAGCTCAGGCGACACGGTCTC-3′	
β-actin (DQ206431.1)	Sense	5′-AGCCATCCTTCTTGGGTATG-3′	138
	Anti-sense	5′-ATACCTGGGAACATGGTGGT-3′	

*T7 promoter sequence (5′-TAATACGACTCACTATAGGGA-3′) has been tagged on to the U-type channel gene sequence for transcribing RNA in dsRNA synthesis.

The 27-mer siRNAs specific to the U-type channel genes were designed using SciTools RNAi Design online software (IDT DNA), and the siRNA sequences are shown in Table 2. Two specific U-type channel siRNA and a control siRNA were purchased from IDT DNA as described previously [21], [50]. TriFECTa control was used as the control siRNA.

**Table 2:** Sequences of siRNAs used in the knockdown study.

Name	Sequence	Oligomers
U-type siRNA 1 (AF484085.1)	5′- UUCAUCAACCAUAACAAGUUUCCAGGA -3′	27
	3′- dA^d^ AGUAGUUGGUAUUGUUCAAAGGUCCU -5′	
U-type siRNA 2 (AF484086.1)	5′- GAUGGUUUGCUUGGCAACUUCUUCCUC -3′	27
	3′- dC^d^ TACCAAACGAACCGUUGAAGAAGGAG -5′	
TriFECTa control	5′- UCACAAGGGAGAGAAAGAGAGGAAGGA -3′	27
	3′- dA^d^ GUGUUCCCUCUCUUUCUCUCCUUCCU -5′	

d N represents a deoxyribonucleotide, and the remaining N represents ribonucleotides.

For dsRNA/siRNA delivery, 10% Listerine anaesthetized snails were injected with µl of dsRNA (3 µg/µl) or siRNA (20 µM) in an area caudal to the buccal mass and dorsal to the central ring ganglia [20], [21]. Aerial respiratory behaviours were observed 3 days post-injection.

### Real-time quantitative polymerase chain reaction (qPCR)

Real-time qPCR was performed using Platinum SYBR Green qPCR SuperMix (Invitrogen). 5 µl of the mix was added to 1 µl of 2.5 µM primers (Table 1) and 0.1 µl cDNA, and topped off with 0.5% diethyl pyrocarbonate-treated water to a final volume of 10 µl. Individual cDNA samples were run in identical triplicates. The reactions were performed in 384-well dishes and run in a Real-Time PCR System (7900HT, Applied Biosystems, ABI) controlled by SDS2.2.1 software, with the cycling parameters of 50°C for 5 min and 95°C for 10 min, followed by 40 cycles of 95°C for 30 seconds and 55°C for 30 seconds followed by a melting curve protocol. The peak of the first-derivative in the melting curve and the shape of the amplification curve were used to assess the quality of the PCR. Ratiometric target (U-type)/control (β-actin) transcript levels were analyzed using the ΔΔC_t_ method [51]. The data were normalized to corresponding naïve control, a reference group, (Ratio  =  (eff_ target gene_)^ΔCTtarget (control-treated)^/(eff_ reference gene_)^ΔCTreference (control-treated)^). A value of one represents no change in the relative mRNA expression levels, with values greater than one representing an increase and values less than one representing a decrease in the relative mRNA expression.

### Primary cell culture

Right pedal dorsal 1 (RPeD1) cells, the pacemaker neurons exhibiting spontaneous firing properties and initiates the respiratory rhythm, were isolated and maintained in culture as previously described [15], [52]. For gene silencing experiments, whole animals were first injected with 2 µl of dsRNA (3 µg/µl) or siRNA (20 µM) 3 days prior to cell isolation. Isolated cells were plated and maintained in culturing media containing 2 µg/ml of dsRNA or 20 nM of siRNA for 12 to 24 hours prior to recording.

### Electrophysiology

Whole-cell patch clamp recordings (ruptured) were performed on cultured RPeD1 neurons, as described previously [53]. The microelectrode pipettes filled with intracellular solution containing (in mM) 29 KCl, 2.3 CaCl_2_, 2 MgATP, 10 HEPES, 11 EGTA, and 0.1 GTPTris (pH 7.6 adjusted with 1 M KOH) were used. Bath solution, consist of saline snail containing (mM): NaCl 51.3, KCl 1.7, CaCl_2_ 4.1, MgCl_2_ 1.5, HEPES 2 (adjusted to pH 7.9 using NaOH), was focally perfused onto the cells with a gravity-driven perfusion system. Signals were recorded and amplified with a personal computer equipped with pClampex 9.2 (Axon Instruments) and MultiClamp 700A connected to Digidata 1322 digitizer, respectively. Data were filtered at 1 kHz (−3 dB) using a 4-pole Bessel filter and digitized at a sampling frequency of 2 kHz. Na^+^-free solution contained (mM) 51.3 N-methyl D-glucamine (NMDG) to substitute Na^+^ ions. Under voltage-clamp mode, leak currents were measured by subtracting the total current traces with the non-linear voltage-dependent currents recorded with a P/4 subtraction protocol using pClampex 9.2 (**Figure 2**). Data were analyzed with Clampfit 9.2 (Axon Instrument) and plotted with Origin Pro v8 (Origin Lab Co., Northhampton, MA, USA). Curve fitting was performed with Origin Pro v8. All the recordings were performed at room temperature (∼22°C).

Under current-clamp mode, conventional sharp electrode recordings were performed to monitor the spontaneous bursting firing activity of RPeD1 cells. Sharp electrodes were filled with saturated K_2_SO_4_ solution (70–80 MΩ) and bath solution with snail saline. Resting membrane potential was measured within 2 minutes after impaling the RPeD1. Input resistance was calculated based on the size of injected hyperpolarizing current and the resulting membrane potential (−70 to −120 mV), following Ohm's law. At the end of each experiment resting membrane potential and electrode resistance were again measured. The spontaneous action potential (AP) frequency and inter-spike intervals were analyzed with Clampfit 9.2 (Axon Instrument). Logarithmic histograms of the inter-spike intervals at bin size 20 were plotted with Clampfit 9.2 to describe the bursting firing pattern. AP amplitude, rise time, decay time, and half width duration were measured Mini Analysis Program ver. 6.01 (Synaptosoft, Decatur, GA, USA).

To test sensitivity of leak current to tetrodotoxin (TTX) block, TTX was added in the bath solution and perfused onto RPeD1 cells. In addition, snail saline containing either Gd^3+^ (10 mM) or low [Ca]_o_ (0.5 mM) was perfused onto the RPeD1 cells to study their effects on the leak current activities and membrane potentials.

### Data analysis and statistics

All data are presented as mean ± S.E.M. Statistical analysis was carried out using OriginPro v8 (Origin Lab Co) or SigmaStat (3.0, Jandel Scientific). Difference between experimental groups was evaluated using a Student's t-test for two groups and one-way analysis of variance (ANOVA) followed by Holm-Sidak post hoc test for multiple experimental groups. Significance was defined by probability level of lower than 0.05 (*P*<0.05).

## Supporting Information

Figure S1 **Partial knockdown of U-type channel reduces inward hyperpolarizing Na^+^ current in RPeD1 neurons.** (A) Representative Na^+^ current generated by subtracting the current obtained in Na^+^ free condition from that in saline from RPeD1 neurons in naive control, control dsRNA, and U-type dsRNA treatments (from the data presented in Figure 5B). (B) Average I_Na_ density-voltage (I–V) relations of naive control (n = 8), control dsRNA (n = 6), and U-type dsRNA (n = 8) treatment. U-type knockdown significantly reduces inward I_Na_ densities at all hyperpolarizing voltages (P<0.05).
(PDF)

Figure S2 **TTX regulates voltage-gated Na^+^ channel of** ***L. stagnalis*** **RPeD1 neuron in a dose-dependent manner.** (A) Representative Na^+^ currents activated from a voltage step from a holding voltage of −50 mV to +10 mV in the absence or presence of various TTX concentrations. Note a residual current that was not blocked by 300 µM of TTX; it is considered TTX-insensitive component. (B) Dose-response curve of the TTX sensitive voltage-gated Na^+^ current. Data are presented as mean ± s.e.m. (n = 3) and the curve was fit with Hill equation. Half-maximal inhibitory concentration (IC_50_) is 23.7 µM.
(PDF)
