# Supplementary material for: A Sodium Leak Current Regulates Pacemaker Activity of Adult Central Pattern Generator Neurons in Lymnaea Stagnalis
Source: PLoS One. 2011 Apr 19;6(4):e18745. doi: 10.1371/journal.pone.0018745 (PMC3079709; doi:10.1371/journal.pone.0018745)
Supplement: Figure S1 — Partial knockdown of U-type channel reduces inward hyperpolarizing Na+ current in RPeD1 neurons. (A) Representative Na+ current generated by subtracting the current obtained in Na+ free condition from that in saline from RPeD1 neurons in naive control, control dsRNA, and U-type dsRNA treatments (from the data presented in Figure 5B). (B) Average INa density-voltage (I–V) relations of naive control (n = 8), control dsRNA (n = 6), and U-type dsRNA (n = 8) treatment. U-type knockdown significantly reduces inward INa densities at all hyperpolarizing voltages (P<0.05). (PDF) [file pone.0018745.s001.pdf]

Supplement materials

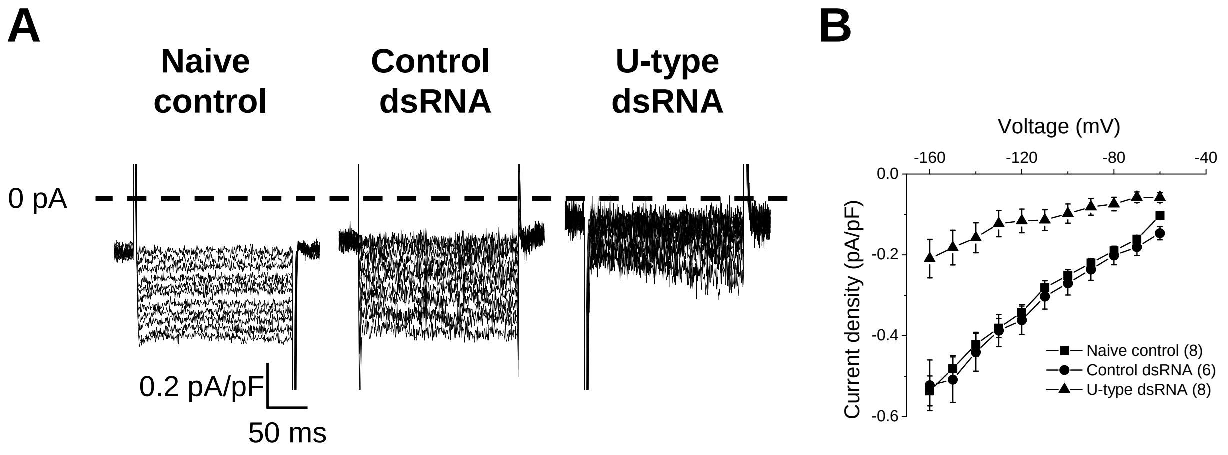

Figure S1. Partial knockdown of U-type channel reduces inward hyperpolarizing Na^+ current in RPeD1 neurons. (A) Representative Na^+ current generated by subtracting the current obtained in Na^+ free condition from that in saline from RPeD1 neurons in naive control, control dsRNA, and U-type dsRNA treatments (from the data presented in Figure 5B). (B) Average I_{Na} density-voltage (I-V) relations of naive control (n = 8), control dsRNA (n = 6), and U-type dsRNA (n = 8) treatment. U-type knockdown significantly reduces inward I_{Na} densities at all hyperpolarizing voltages ($P < 0.05$).
