# Supplementary material for: A Sodium Leak Current Regulates Pacemaker Activity of Adult Central Pattern Generator Neurons in Lymnaea Stagnalis
Source: PLoS One. 2011 Apr 19;6(4):e18745. doi: 10.1371/journal.pone.0018745 (PMC3079709; doi:10.1371/journal.pone.0018745)
Supplement: Figure S2 — TTX regulates voltage-gated Na+ channel of L. stagnalis RPeD1 neuron in a dose-dependent manner. (A) Representative Na+ currents activated from a voltage step from a holding voltage of −50 mV to +10 mV in the absence or presence of various TTX concentrations. Note a residual current that was not blocked by 300 µM of TTX; it is considered TTX-insensitive component. (B) Dose-response curve of the TTX sensitive voltage-gated Na+ current. Data are presented as mean ± s.e.m. (n = 3) and the curve was fit with Hill equation. Half-maximal inhibitory concentration (IC50) is 23.7 µM. (PDF) [file pone.0018745.s002.pdf]

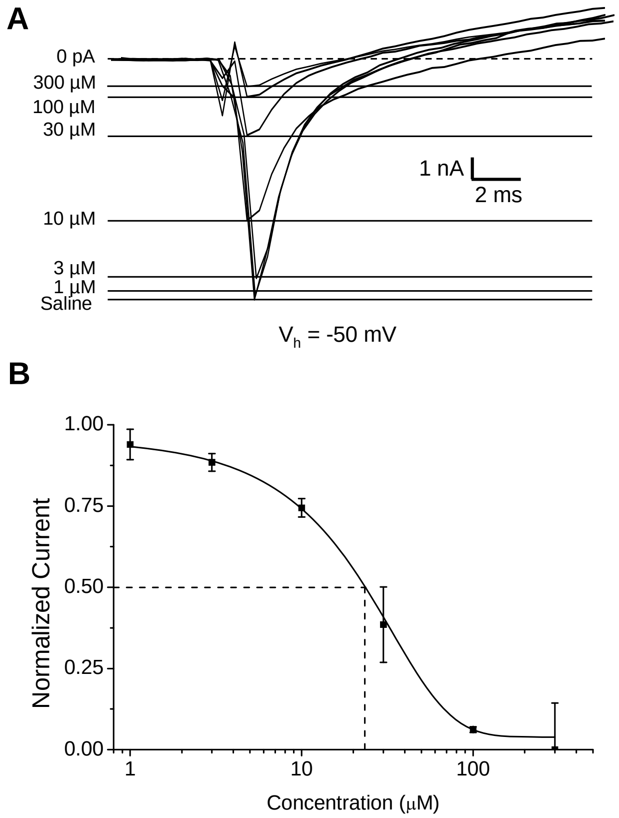


**Figure S2. TTX regulates voltage-gated $Na^+$ channel of *L. stagnalis* RPeD1 neuron in a dose-dependent manner.** (A) Representative $Na^+$ currents activated from a voltage step from a holding voltage of -50 mV to +10 mV in the absence or presence of various TTX concentrations. Note a residual current that was not blocked by 300 µM of TTX; it is considered TTX-insensitive component. (B) Dose-response curve of the TTX sensitive voltage-gated $Na^+$ current. Data are presented as mean ± s.e.m. (n = 3) and the curve was fit with Hill equation. Half-maximal inhibitory concentration ($IC_{50}$) is 23.7 µM.
